# Cost Savings Associated With Fully Automated Digital Cognitive Behavioral Therapy for Insomnia Disorder (SleepioRx): A Matched Control Study of US Patients

**DOI:** 10.36469/001c.146434

**Published:** 2025-11-13

**Authors:** Christopher B. Miller, Danielle Bradley, Shana Hall, Helen Hayes, Sulayman Chowdhury, Chris Sampson

**Affiliations:** 1 Big Health, London, UK; 2 Big Health, San Francisco, California, USA; 3 Office Of Health Economics (OHE), London, UK

**Keywords:** digital CBT-I, cost savings, digital treatments, insomnia, real-world evidence, SleepioRx

## Abstract

**Background:**

Insomnia affects up to one-third of US adults and is a significant health challenge with an estimated economic burden of up to $100 billion annually. Cognitive behavioral therapy (CBT) for insomnia (CBT-I) is the recommended first-line treatment, but access is limited due to a shortage of trained therapists. Digital CBT-I offers an effective alternative that may enhance accessibility and reduce higher healthcare costs associated with insomnia.

**Objective:**

To evaluate the US healthcare cost-savings of digital CBT-I compared with standard-of-care control.

**Methods:**

A retrospective difference-in-differences analysis compared 1-year preinitiation and post-initiation healthcare costs for 11 027 individuals receiving SleepioRx (FDA-cleared digital CBT treatment for insomnia disorder) compared with 1:1 exact matched controls with insomnia receiving standard care (n = 10 770). Commercial and Medicare claims were adjusted for comorbidities, index year, and baseline utilization.

**Results:**

Digital CBT-I was associated with statistically significant mean annual total cost savings of 2083(951508-$2657, *P* < .001) per person, equating to a 42% reduction in costs with SleepioRx relative to matched controls who received standard of care (medications for insomnia).

**Discussion:**

Digital CBT-I was associated with substantial cost savings for payers. The integration of guideline-concordant treatment through digital delivery into standard care pathways offers a promising strategy to address the clinical and economic challenges of insomnia, supporting more efficient resource allocation.

**Conclusions:**

Findings suggest that implementing digital CBT-I at scale may lead to decreased costs for healthcare payers, relative to the current standard of care, while improving access to effective insomnia treatment.

## INTRODUCTION

Insomnia affects 10% to 35% of US adults,^1,2^ making it the most prevalent sleep disorder and one of the top three most common mental health conditions in primary care.^3–5^ This widespread condition poses substantial economic burden and public health challenges. Median healthcare costs are 4 to 6 times greater for those with insomnia than for those without,^6^ with sleep-promoting hypnotic medications increasing annual costs by 80%.^7,8^ Second-line hypnotic treatments often fail to address underlying insomnia symptoms and their longer-term clinical benefit is not known.^9,10^ Hypnotics are associated with poor cost-benefit profiles and substantial risks, particularly for older adults; these include more than a 2-fold higher risk of falls, a 15-fold higher mortality rate, and over double the monthly healthcare costs.^11,12^

Despite guidelines recommending cognitive behavioral therapy (CBT) for insomnia (CBT-I) as the first-line treatment,^9,13^ the US healthcare system demonstrates structural barriers to cost-effective delivery. Nationwide, fewer than 800 clinicians are certified to provide CBT-I.^14^ Consequently, only 3% of Medicare patients receive CBT-I as their sole treatment.^15^ Access barriers extend beyond provider availability to fundamental reimbursement challenges. Only 56% of surveyed CBT-I providers accept any insurance coverage, with virtually no Medicare acceptance (1 provider nationwide). Economic barriers are further evidenced by initial visit costs averaging $260, subsequent sessions costing $227, and provider wait times averaging more than 51 days.^16^ Unable to obtain guideline-concordant and cost-effective care, patients frequently receive second-line and off-label medications, including trazodone, one of the most common medications used for insomnia in primary care.^17^ This misalignment between evidence-based guidelines and clinical practice results in Medicaid patients receiving predominantly zolpidem and off-label trazodone prescriptions.^18^ This pattern exemplifies the clinical and economic inefficiency of current insomnia care delivery.

Against this backdrop of access barriers and costs, we conducted an economic evaluation of SleepioRx, an FDA-cleared digital mental health treatment delivering fully automated CBT-I for those at least 18 years of age. SleepioRx has demonstrated proven clinical efficacy, with 76% of patients achieving healthy sleep in controlled trials,^19^ and effectiveness in improving functional health and psychological well-being across diverse patient populations.^20^ Economic modeling has demonstrated digital CBT-I as a cost-effective treatment option compared with individual and group CBT and pharmacotherapy, generating a positive net monetary benefit over 6 months,^21^ while clinical trial evidence has shown the statistically significant gains in quality-adjusted life-years (QALYs).^22^ Gains in health status may last 3 to 4 years after SleepioRx treatment start compared with standard-of-care control.^23^ Cost savings have been demonstrated in UK clinical settings, with projected national savings of £20 million in the first year through reduced primary care costs and reduced hypnotic prescribing in England.^24^ However, real-world economic impact on US healthcare spending has not been quantified at a population scale, reaching those with insomnia across diverse locations and clinical settings.

This analysis assessed SleepioRx’s cost-saving potential from a US payer perspective using claims data, addressing whether access to effective digital CBT-I may reduce the healthcare burden and associated costs of insomnia.

## METHODS

### Study Design and Framework

This retrospective observational cohort study assessed the economic impact of digital CBT-I (SleepioRx) compared with standard care for insomnia. The study was designed from a US healthcare payer perspective, encompassing both commercial insurance and Medicare claims to reflect real-world healthcare utilization and costs across diverse patient populations. The study employed a quasi-experimental design comparing individuals who used SleepioRx treatment with matched controls who did not receive the digital intervention and used standard of care for their insomnia.

### Data Source and Study Population

**Data source**: The study utilized de-identified administrative claims data from US commercial insurance and Medicare programs, spanning 2013 to 2023. Claims data were provided by Kythera, a national claims data aggregator, and underwent validation through an independent Privacy Expert Determination Certification Report to ensure HIPAA (Health Insurance Portability and Accountability Act) compliance. Individual-level data linkage between healthcare claims and SleepioRx utilization records was performed using Datavant’s HIPAA-compliant de-identified privacy-preserving record linkage technology.

**Treatment cohort definition**: The treatment cohort comprised individuals who initiated SleepioRx during the study period. All participants were required to be aged 18 years or older at the index date, defined as the first date of SleepioRx initiation. Participants maintained continuous health plan enrollment for at least 12 months both pre-index and post-index to enable comprehensive cost analysis across baseline and follow-up periods.

**Control population identification**: The control population was identified from a nationally representative pool of approximately 1.3 million claimants who received standard of care for insomnia but did not use SleepioRx. Controls were first identified from a sample of patients with insomnia diagnoses (*International Classification of Diseases, Tenth Revision* [ICD-10] diagnostic codes) or insomnia-indicated prescriptions, with the index event defined by prescription fill only. These diagnostic and medication criteria are consistent with established methodologies for insomnia detection in claims data.^6,15^ The data structure used an index date to allow for pre-post comparison of SleepioRx to the control group. For controls, the index date was defined as the earliest qualifying prescription fill date. We used the prescription fill date because there was a very high overlap rate for those managed with insomnia medications and those who had an insomnia diagnosis code. Controls were also required to meet both age and continuous enrollment requirements.

**Inclusion and exclusion criteria**: To enhance real-world generalizability, minimal exclusions were applied. Individuals were excluded only for insufficient baseline or follow-up claims data for cost calculations. No exclusions were made based on comorbidities or concurrent treatments, ensuring a representative patient population.

**Ethics and compliance**: This study utilized de-identified claims data certified through independent expert determination under HIPAA regulations. Under federal regulations (45 CFR § 46.104), analysis of such data does not constitute US human subjects research and did not require Institutional Review Board approval.

### Outcomes, Data Processing, Matching and Statistical Analysis

**Outcomes**: The primary outcome was change in total healthcare costs from baseline (12 months pre-index) to follow-up (12 months post-index) between treatment and control groups. Total healthcare costs encompassed medical claim costs (inpatient services, outpatient services, emergency department visits) and pharmacy claim costs (all prescription medications). Secondary outcomes included individual cost components analyzed separately as well as healthcare utilization measures. All costs were calculated using the “allowed” amount from claims data and inflated to 2023 US dollars using the Medical Care Consumer Price Index to ensure comparability across the study period.

**Data processing and preparation:** The raw prescription (Rx) and medical (Mx) claims data sets underwent systematic cleaning and validation procedures. Duplicate records were removed, participants were matched, and unique index dates were established based on SleepioRx enrollment or relevant earliest prescription dates for insomnia medications. The Rx data was filtered for final transactions, inflated to 2023 costs, categorized, and collapsed to monthly levels. The Mx data were aggregated per claim, inflated, and categorized by ICD-10 codes to capture comorbidities. Both data sets were merged, transformed into annualized patient-level data, and trimmed for analysis, ensuring alignment across demographics, costs, and time horizons. For missing values, we made two assumptions. First, missing post-index claims (ie, no observed healthcare encounters) were coded as zero cost, conservatively reflecting that nonobservation in claims data equates to no healthcare utilization. Second, for observed Mx claims with missing final paid amounts, submitted charges were more complete but typically exceeded final payments. Using a larger claims data set from 2023 provided by Kythera, we calculated average final payments stratified by claim type, bill code, place of service, and procedure codes, and imputed missing payment values using corresponding averages. All analyses were conducted using observed data without further adjustment for missing activity data, given the scale of the data set and sample size. Because patients were matched on pre-index characteristics, the difference-in-difference analysis proceeded without adjustment for time-varying covariates. All data cleaning and processing was conducted using SQL.

**Exact matching and final study population**: One-to-one exact matching (without replacement) was employed to balance treatment and control groups on observable characteristics. Treatment individuals were matched to controls based on demographic characteristics (age, gender, insurance type, state of residence), pre-existing comorbidities assessed through claims (diabetes, cardiovascular disease, chronic obstructive pulmonary disease, irritable bowel syndrome, cancer, asthma, depression, anxiety, insomnia), and index year of intervention initiation to control for temporal trends. Following matching, the final study population comprised 11 027 individuals in the treatment cohort and 10 770 unique individuals in the control cohort (some controls were matched to multiple-treated individuals). All matching was performed using SQL.

**Statistical analysis**: Between-group comparisons employed difference-in-differences regression analyses to estimate the effect of SleepioRx on healthcare costs. The model compared change in costs from baseline to follow-up periods between treatment and matched control groups. This method controlled for baseline imbalances between groups and allowed for adjustment of baseline matching variables (comorbidities and index year), and temporal trends to control for any residual variation between matched groups. The regression models also adjusted for baseline differences in healthcare utilization, including prescriptions. All statistical analyses employed ordinary least squares regression models with robust standard errors using R (version 4.3.1)^25^ on a Wayfinder/Databricks runtime cluster. Healthcare cost outliers were retained without winsorizing as they may represent legitimate clinical complexity and high-utilization patterns in patients with insomnia.^26^

## RESULTS

### Cohort Population Sample

The final study population consisted of 21 797 participants across treatment and matched control groups. Demographic and clinical characteristics were well-balanced between groups, with participants averaging 44 years of age and 66% female representation (**Table 1**).

**Table 1.** Demographic and Clinical Characteristics

**Measure**	**Overall (n = 21 797)**	**SleepioRx (n = 11 027)**	**Control (n = 10 770)**
Age, y			
Average	44	44	44
Range	18-67	18-67	18-67
Gender, %			
Male	34	34	34
Female	66	66	66
Top 5 most frequent comorbidities (from Mx ICD-10 code data) (%)			
Cardiovascular	12	12	12
Anxiety	7	7	7
Depression	4	4	4
Diabetes	3	3	3
Asthma	2	2	2
Index year, %			
2017	3	3	3
2018	10	10	10
2019	41	41	41
2020	20	20	20
2021	20	20	20
2022	6	6	6
Location,^a^ %			
Northeast	14	14	14
Southeast	35	35	35
Midwest	24	24	24
Southwest	8	8	8
West	18	18	18
Average Mx utilization (No. of claims within pre-period)	6	5	7
Average Rx utilization (No. of filled prescriptions within pre-period including refills)	22	14	29

Abbreviations: ICD-10, *International Classification of Disease, Tenth Revision*; Mx, medical; Rx, prescription.

^a^Classified as Northeast (9 states): CT, ME, MA, NH, RI, VT, NJ, NY, PA, DE; Southeast (12 states): AL, AR, FL, GA, KY, LA, MS, NC, SC, TN, VA, WV; Midwest (12 states): IL, IN, IA, KS, MI, MN, MO, NE, ND, OH, SD, WI; Southwest (4 states): AZ, NM, OK, TX; West (13 states): AK, CA, CO, HI, ID, MT, NV, OR, UT, WA, WY.

**Economic impact on healthcare claim costs**: In the 12-month baseline period, mean (SD) annual total healthcare costs were $4985 ($13 292) for the SleepioRx group and $8444 ($19 195) for the control group. Despite exact matching, baseline cost differences remained between groups (**Supplementary Table S1**). This disparity likely reflects unmeasured differences in disease severity, treatment pathways, or healthcare-seeking behavior that matching on observable characteristics could not fully address.

**Figure 1.**
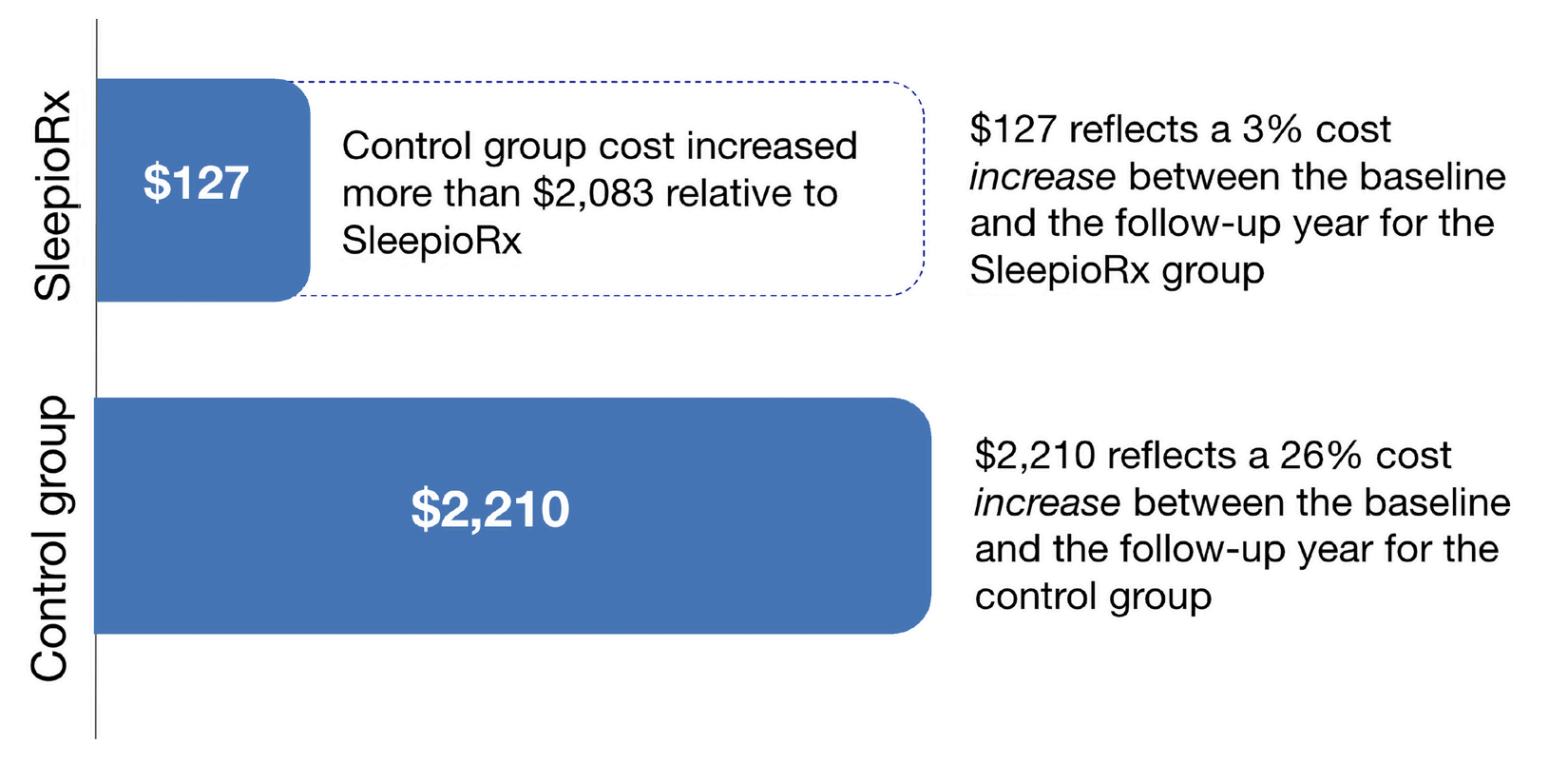
Change in Average Annual Total Healthcare Costs from Baseline to Follow-up Period by Group Change in average annual total healthcare costs per person comparing 1 year posttreatment to 1 year pretreatment by group. SleepioRx users demonstrated $2083 greater cost reduction (42%) compared with controls (unadjusted presentation).

**Overall cost analysis:** The difference-in-differences effect on overall healthcare expenditure revealed a $2083 average per person per year cost reduction for those who had used digital CBT-I relative to matched controls. This represents a 42% relative reduction in total healthcare costs. **Figure 1** displays these unadjusted descriptive results. This effect was statistically significant in the unadjusted regression analysis (95% CI, $1443-$2722: *P* < .001) (**Supplementary Table S2**). Similar results were found when adjusted for both utilization (**Supplementary Table S3**), and for utilization, year and comorbidities (**Supplementary Table S4**).

### Cost Component Analysis

**Figure 2.**
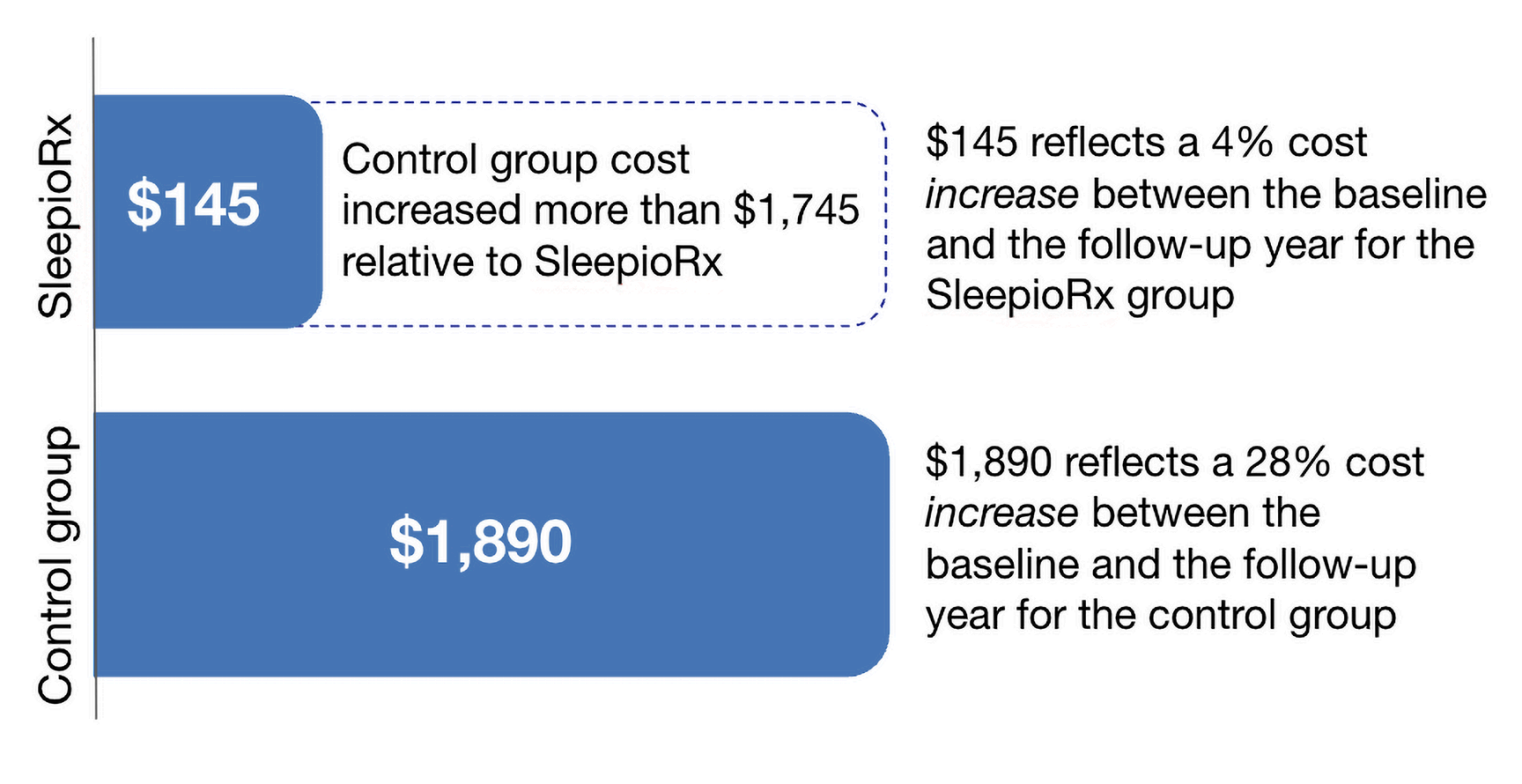
Change in Average Annual Pharmacy Costs from Baseline to Follow-up Period by Group Change in average annual pharmacy costs per person comparing 1 year posttreatment to 1 year pretreatment by group. SleepioRx users demonstrated $1745 greater cost reduction (46%) compared with controls (unadjusted presentation).

**Pharmacy costs:** The difference-in-differences effect found a $1745 average per person per year cost reduction for those with digital CBT-I compared with controls, representing a 46% relative reduction in pharmacy costs. **Figure 2** reports these unadjusted descriptive results. The effect was statistically significant in the unadjusted regression analysis (95% CI, $1211-$2280; *P* < .001) (**Supplementary Table S5**). Results were consistent when adjusted for utilization (**Supplementary Table S6**), and for utilization, year and comorbidities (**Supplementary Table S7**).

**Figure 3.**
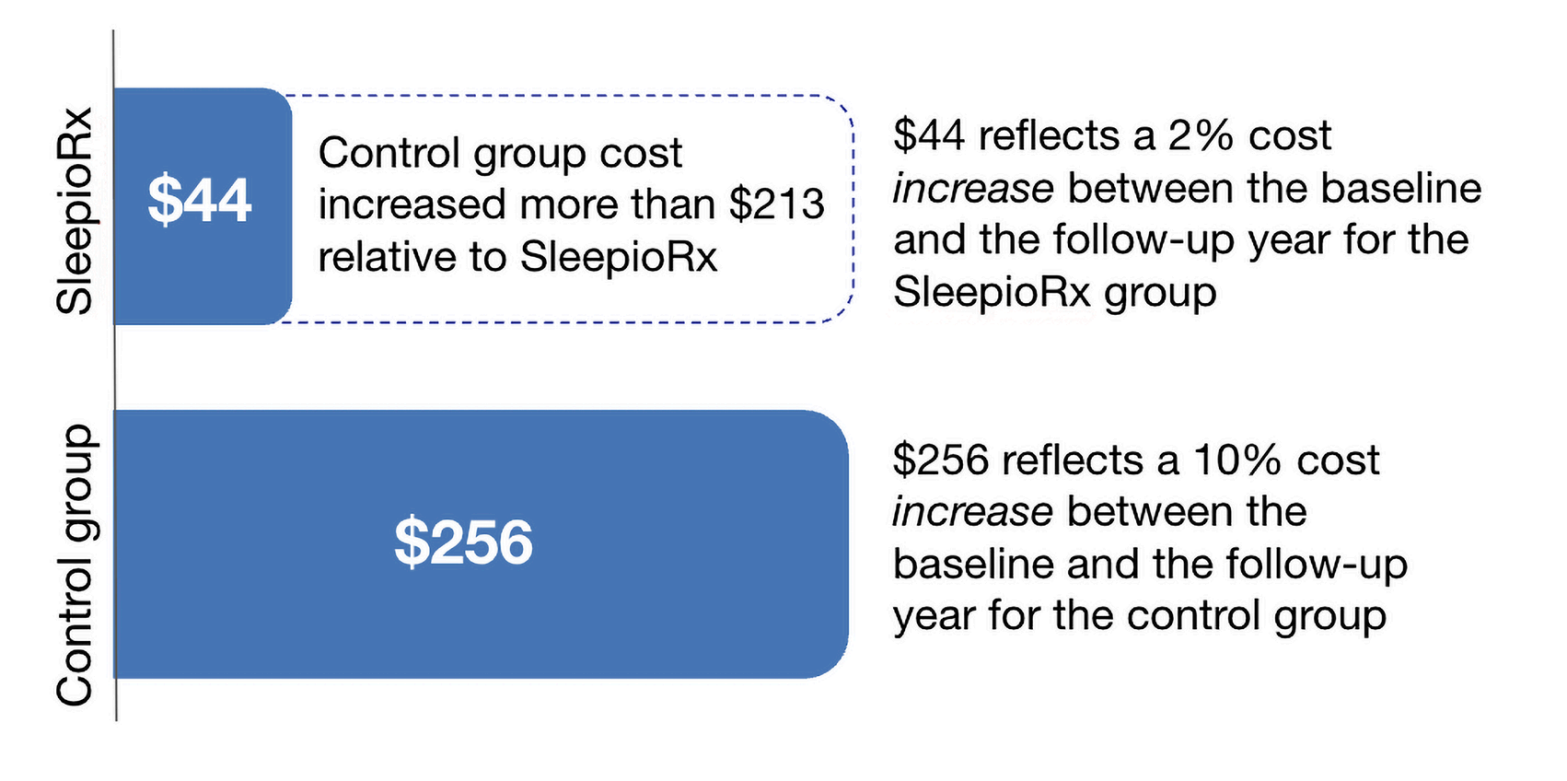
Change in Average Annual Medical Costs from Baseline to Follow-Up Period By Group Change in average annual medical costs per person comparing 1 year posttreatment to 1 year pretreatment by group. SleepioRx users demonstrated $213 greater cost reduction (10%) compared with controls, though this difference was not statistically significant (unadjusted presentation).

**Medical service costs**: For medical claim costs, the difference-in-differences effect observed a reduction of $213 per person annually among SleepioRx patients compared with controls. This represents a 10% relative reduction. **Figure 3** shows these unadjusted descriptive results. This effect was not statistically significant in the unadjusted regression analysis (95% CI, -$681 to $257; *P* = .375) (**Supplementary Table S8**). Trends toward statistical significance (*P* range, .157-.161) were observed when adjusted for utilization (**Supplementary Table S9**), and for utilization, year, and comorbidities (**Supplementary Table S10**). Both adjusted results found a 15% relative reduction in costs (trend savings of $316-$321).

**Figure 4.**
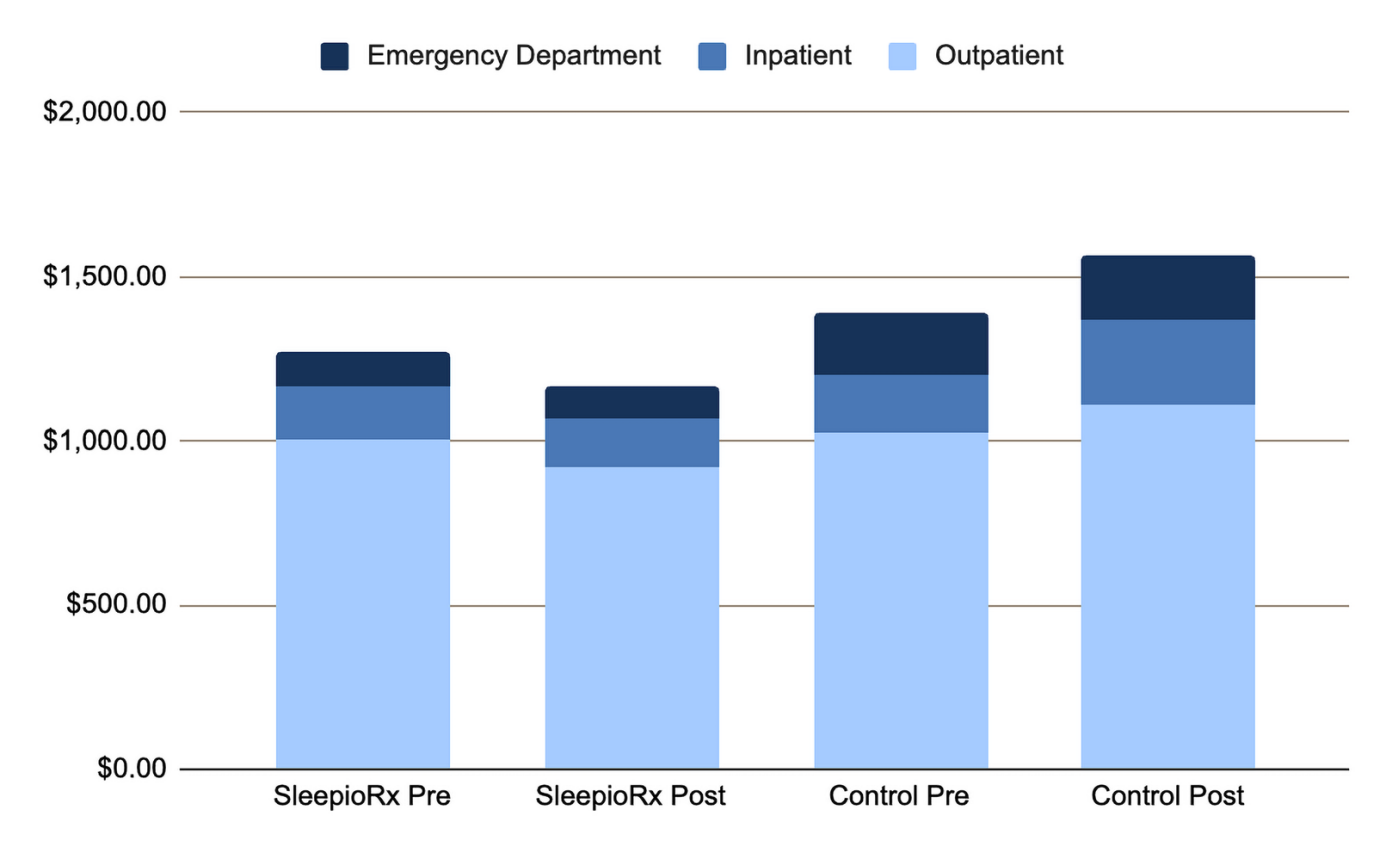
Annual Medical Claim Costs by Service Type, Displaying Baseline and Follow-up Periods by Group Annual average medical claim costs per person by service type, comparing 1 year posttreatment to 1 year pretreatment by group (unadjusted presentation).

**Medical service categories**: Cost changes varied across medical service types (**Figure 4**), which reports the descriptive breakdown of medical claim costs by service category, comparing baseline and follow-up periods between treatment groups. Specifically, the digital CBT-I group’s costs were 65% lower for inpatient care, 16% lower for outpatient services, and 14% lower for emergency department costs.

## DISCUSSION

In this real-world analysis of 21 797 US adults, SleepioRx, an FDA-cleared digital CBT-I treatment, generated mean first-year savings of $2083 per person (42% reduction) compared with matched controls who received standard care, with concordant unadjusted and adjusted estimates confirming robustness.

Overall pharmacy expenditures accounted for the majority of savings ($1745; 46% reduction; *P* < .001); this may reflect reduced reliance on sleep-promoting hypnotics that are associated with up to 85% higher overall healthcare costs and elevated risks of falls, fractures, and mortality, particularly in older adults.^7,11^ Although medical claim costs fell by 10% to 15% (not significant; *P* range, .157-.161), consistent downward trends across inpatient, outpatient, and emergency department services suggest broader utilization benefits that may become statistically significant with longer follow-up. These findings extend national estimates in England of first-year savings from primary care settings of £20 million^24^ to a diverse US population spanning commercial and Medicare plans and strengthens the evidence for digital CBT-I’s cost benefits at scale.

Given that insomnia affects 10% to 35% of US adults and drives 4 to 6 times higher annual costs,^2,6^ these findings underscore digital CBT-I’s potential to alleviate a substantial economic burden at a population scale, reaching those who may have difficulty accessing therapist delivered CBT-I.^14^ Karlin et al further reported that individuals with insomnia incur nearly $1000 higher mean monthly costs, and more than $1260 when comorbidities are present.^6^ Compared with therapist-delivered CBT-I (~$1000 per 6-session course)^21^ and only 3% uptake in Medicare patients,^15^ digital CBT-I offers superior budget impact and scalability amid a national shortage of certified CBT-I providers.^14^ Results complement existing clinical trial results, which have previously shown that improvements in sleep with digital CBT-I explain improvements in functional health, and psychological well-being.^20^ Health improvements have been found to persist for up to 3 to 4 years from treatment start.^23^ Beneficial effects have also been observed in workplace productivity.^27^

We acknowledge baseline cost disparities ($4985 vs $8444) that likely reflect higher-cost trajectories among standard-of-care control patients. Increased costs have been found to precede insomnia diagnosis by at least 6 months.^7^ The difference-in-differences framework was expressly chosen to isolate within-patient cost changes over time and mitigate time-invariant unobserved confounding. Sensitivity analyses varying covariate sets including index year, medical and medication utilization adjustments produced very similar estimates, underscoring robustness and strengthening confidence in the matching process effectively balancing treatment and control groups on observable characteristics.

Strengths include the large, heterogeneous sample, minimal exclusions, and rigorous quasi-experimental design. Limitations encompass potential unmeasured confounding and residual selection bias due to factors influencing treatment assignment such as patient motivation and provider channeling preferences, a 1-year time horizon focused on a payer perspective, lack of direct clinical outcome measures in administrative claims data, and absence of comparison with therapist-delivered CBT. Such a direct comparison may be potentially impractical given limited provider availability,^14,16^ relative to the large number who received digital CBT-I (>10 000). This study evaluated those who likely received medications only for treatment of their insomnia; this is in line with standard care practice, where the vast majority of patients treated (>90%) receive medications for their sleep.^15^

The validity of a difference-in-differences design is reliant on the parallel trends assumption that digital CBT-I participants and controls would have experienced similar cost trajectories in the absence of the intervention. This assumption is fundamentally unknowable. However, our exact matching procedure on baseline characteristics supports the plausibility of parallel trends, as well-matched comparison groups typically satisfy this assumption.^28^ Due to the pre-post design, however, we were unable to formally test the parallel trends assumption statistically, the quality of our matching strengthens confidence in the comparability of our treatment and control groups. Consequently, we present cost savings in associative terms, relative to matched controls receiving standard care, rather than as definitive causal effects.

Future studies should assess multi-year cost trajectories, to better understand how costs may change over time, integrate patient-reported outcomes found within chart data, and evaluate digital CBT-I impact in subgroups (eg, high insomnia severity, comorbidity, older adults), and insomnia subtypes. Integrated health economic analyses combining direct healthcare costs, clinical severity (Charlson Comorbidity Index) and workplace productivity, may help better capture both payer and employer perspectives simultaneously using novel data linkage techniques.

## CONCLUSIONS

Implementation of digital CBT-I reduced first-year total healthcare costs by $2083 per patient (42% reduction) compared with standard of care insomnia pharmacotherapy, driven by a 46% reduction in pharmacy expenditures and downward trends across medical services. As insomnia prevalence and medication costs rise, digital CBT-I provides a scalable, guideline-concordant treatment that aligns clinical benefits with economic efficiency. These results support payer adoption of SleepioRx to mitigate the high clinical and financial burden of insomnia.

### Note

SleepioRx is a digital therapeutic intended for the treatment of chronic insomnia/insomnia disorder as an adjunct to usual care in patients aged 18 and older. SleepioRx is a prescription device delivering Cognitive Behavioral Therapy for Insomnia (CBT-I) and can be made available on the order of a licensed healthcare provider. Patients should read the instructions for use for full information.

### Disclosures

This work was supported by Big Health Inc., the company that develops and commercializes SleepioRx.

## Supplementary Material

Online Supplementary Material
