# Supplementary material for: Cost Savings Associated With Fully Automated Digital Cognitive Behavioral Therapy for Insomnia Disorder (SleepioRx): A Matched Control Study of US Patients
Source: J Health Econ Outcomes Res. 2025 Nov 13;12(2):193–9. doi: 10.36469/001c.146434 (PMC12619666; doi:10.36469/001c.146434)
Supplement: Online Supplementary Material [file jheor_2025_12_2_146434_310297.pdf]

Journal of Health Economics and Outcomes Research

Online Supplementary Material

Cost Savings Associated With Fully Automated Digital Cognitive Behavioral Therapy for Insomnia Disorder: A Matched Control Study of US Patients. *JHEOR*. 2025;12(2):193-199. doi:10.36469/jheor.2025.146434

Table S1: Descriptive Statistics

Table S2: Unadjusted Regression Model: Total Costs

Table S3: Utilization Adjusted Regression Model: Total Costs

Table S4: Utilization, Year, and Comorbidities-Adjusted Regression Model: Total Costs

Table S5: Unadjusted Regression Model: Prescription Costs

Table S6: Utilization-Adjusted Regression Model: Prescription Costs

Table S7: Utilization, Year, and Comorbidities-Adjusted Regression Model: Prescription Costs

Table S8: Unadjusted Regression Model: Medical Costs

Table S9: Utilization-Adjusted Regression Model: Medical Costs

Table S10: Utilization, Year, and Comorbidities–Adjusted Regression Model: Medical Costs

This supplementary material has been provided by the authors to give readers additional information about their work.

This is an open-access article distributed under the terms of the Creative Commons Attribution 4.0 International License (CCBY-4.0). View this license's legal deed at http://creativecommons.org/licenses/by/4.0 and legal code at http://creativecommons.org/licenses/by/4.0/legalcode for more information.

Table S1. Descriptive Statistics

	Control		Treatment	
	**Pre**	**Post**	**Pre**	**Post**
Total costs, $				
Average	8444.208	10 653.99	4984.913	5112.054
Minimum	0	0	0	0
Maximum	1 149 084	550 352.8	479 958.7	853 457.3
SD	19 194.83	19 339.7	13 292.13	15 376.61
Medical costs (total), $				
Average	2519.5	2775.901	2129.615	2173.44
Minimum	0	0	0	0
Maximum	1 119 545	207 000.8	329 869.5	191 402.6
SD	15 153.87	8225.463	7482.901	6773.682
Prescription costs (total), $				
Average	6761.043	8651.325	3786.757	3931.612
Minimum	0	0	0	0
Maximum	417 797.3	550352.8	479 958.7	849 364
SD	13 729.04	17 159.43	11 441.26	13 940.95
Medical costs (inpatient), $				
Average	172.3858	264.6725	160.3198	149.2827
Minimum	0	0	0	0
Maximum	56 458.31	202 364.7	65 702.52	83 113.95
SD	1696.993	3633.471	1644.997	1715.175
Medical costs (outpatient), $				
Average	1027.229	1106.977	1005.209	920.155
Minimum	0	0	0	0
Maximum	106 011.5	101 105	329 712.8	74 881.83
SD	2969.144	3043.129	5162.134	2753.525
Medical costs (emergency room), $				
Average	188.1196	191.3063	108.4343	96.21606
Minimum	0	0	0	0
Maximum	33 276.17	36 094.77	17 515.91	19 214.44
SD	908.7025	962.7605	573.7834	528.8491
Baseline age, y				
Average	43.93519	43.93519	43.88963	43.88963
Minimum	18	18	18	18
Maximum	67	67	67	67
SD	11.94212	11.94212	11.94588	11.94588
Baseline male (binary)				
Average	0.34234	0.34234	0.342523	0.342523
Minimum	0	0	0	0
Maximum	1	1	1	1
SD	0.474515	0.474515	0.474575	0.474575
Average medical utilization	7.19545	7.19545	4.513467	4.513467
Average prescription utilization	29.04503	29.04503	14.44201	14.44201
N	10 770	10 770	11 027	11 027

Table S2. Unadjusted Regression Model: Total Costs

Model	Response_Variable	Observations (Nx2)	Adjusted_R_Squared		95% CI Lower Bound	95% CI Upper Bound
Unadjusted	total_costs	43594	0.019241			
term	estimate	robust_se	t_stat	p_value		
(Intercept)	8444.208	184.9593	45.65442	0	8081.688	8806.728
sleepio_dummy	−3459.29	224.1261	−15.4346	0	−3898.58	−3020.01
post_insomnia_sleepio_strict	2209.782	262.5608	8.416268	0	1695.163	2724.402
DiD effect	−2082.64	326.1943	−6.38466	1.72E−10	−2721.98	−1443.3

Abbreviations: CI, confidence interval; DiD, difference-in-differences.

Table S3. Utilization-Adjusted Regression Model: Total Costs

Model	Response_Variable	Observations (Nx2)	Adjusted_R_Squared		95% CI Lower Bound	95% CI Upper Bound
Unadjusted	total_costs	43594	0.207181			
term	estimate	robust_se	t_stat	p_value		
(Intercept)	−259.314	186.4891	−1.3905	0.164376	−624.832	106.205
sleepio_dummy	694.9007	192.6151	3.607717	0.000309	317.3752	1072.426
post_insomnia_sleepio_strict	2209.782	234.6102	9.418952	0	1749.946	2669.618
baseline_mx_utilization	236.9283	21.72005	10.90827	0	194.357	279.4996
baseline_rx_utilization	240.9609	6.494657	37.1014	0	228.2313	253.6904
DiD effect	−2082.64	293.226	−7.10251	1.23E−12	−2657.36	−1507.92

Abbreviations: CI, confidence interval; DiD, difference-in-differences.

Table S4. Utilization, Year, and Comorbidities-Adjusted Regression Model: Total Costs

Model	Response_Variable	Observations (Nx2)	Adjusted_R_Squared		95% CI Lower Bound	95% CI Upper Bound
Unadjusted	total_costs	43594	0.207181			
term	estimate	robust_se	t_stat	p_value		
(Intercept)	−240478	132606	−1.81348	0.069759	−500386	19430.08
sleepio_dummy	638.9829	187.7149	3.404008	0.000664	271.0617	1006.904
post_insomnia_sleepio_strict	2209.782	234.4548	9.425197	0	1750.251	2669.314
baseline_anxiety	−907.017	396.9451	−2.28499	0.022313	−1685.03	−129.004
baseline_insomnia	264.7445	434.9271	0.60871	0.542717	−587.713	1117.202
baseline_diabetes	2728.033	823.0282	3.314629	0.000918	1114.898	4341.169
baseline_cardiovascular	791.9746	313.7147	2.524506	0.011586	177.0938	1406.855
baseline_depression	−1810.26	467.1742	−3.87492	0.000107	−2725.92	−894.6
baseline_copd	−277.624	3078.387	−0.09018	0.92814	−6311.26	5756.016
baseline_asthma	−150.797	1317.46	−0.11446	0.908873	−2733.02	2431.424
baseline_ibs	818.2517	2221.383	0.368352	0.712611	−3535.66	5172.162
baseline_mx_utilization	232.2908	23.98943	9.683052	0	185.2716	279.3101
baseline_rx_utilization	237.882	6.270039	37.93947	0	225.5927	250.1712
year_index_date	118.9689	65.64986	1.812172	0.06996	−9.70487	247.6426
DiD effect	−2082.64	292.9041	−7.11032	1.16E−12	−2656.73	−1508.55

Abbreviations: CI, confidence interval; DiD, difference-in-differences.

Table S5. Unadjusted Regression Model: Prescription Costs

Model	Response_Variable	Observations	Adjusted_R_Squared		95% CI Lower Bound	95% CI Upper Bound
Unadjusted	sum_rx_cost	43594	0.020075			
term	estimate	robust_se	t_stat	p_value		
(Intercept)	6761.043	132.2916	51.10714	0	6501.752	7020.335
sleepio_dummy	−2974.29	171.3831	−17.3546	0	−3310.2	−2638.38
post_insomnia_sleepio_strict	1890.281	211.7558	8.926704	0	1475.24	2305.322
DiD effect	−1745.43	272.6473	−6.40177	1.54E−10	−2279.81	−1211.04

Abbreviations: CI, confidence interval; DiD, difference-in-differences.

Table S6. Utilization-Adjusted Regression Model: Prescription Costs

Model	Response_Variable	Observations	Adjusted_R_Squared		95% CI Lower Bound	95% CI Upper Bound
Utilization-adjusted	sum_rx_cost	43594	0.207599			
term	estimate	robust_se	t_stat	p_value		
(Intercept)	−527.374	157.0143	−3.35877	0.000783	−835.123	−219.626
sleepio_dummy	657.2444	149.3123	4.401811	1.07E−05	364.5923	949.8965
post_insomnia_sleepio_strict	1890.281	185.9994	10.16283	0	1525.722	2254.84
baseline_mx_utilisation	35.14123	10.65678	3.297546	0.000975	14.25393	56.02852
baseline_rx_utilisation	242.2294	5.942282	40.7637	0	230.5825	253.8763
DiD effect	−1745.43	245.0192	−7.12363	1.05E−12	−2225.66	−1265.19

Abbreviations: CI, confidence interval; DiD, difference-in-differences.

Table S7. Utilization, Year, and Comorbidities-Adjusted Regression Model: Prescription Costs

Model	Response_Variable	Observations	Adjusted_R_Squared		95% CI Lower Bound	95% CI Upper Bound
Adjusted	sum_rx_cost	43594	0.209228			
term	estimate	robust_se	t_stat	p_value		
(Intercept)	-299000	106875.8	-2.79764	0.005148	-508477	-89523.7
sleepio_dummy	616.4154	148.429	4.15293	3.28E-05	325.4945	907.3362
post_insomnia_sleepio_strict	1890.281	185.9584	10.16507	0	1525.803	2254.76
baseline_anxiety	-758.987	344.9115	-2.20053	0.02777	-1435.01	-82.9604
baseline_insomnia	533.6795	395.8905	1.348048	0.177643	-242.266	1309.625
baseline_diabetes	2523.569	763.6258	3.30472	0.000951	1026.863	4020.276
baseline_cardiovascular	224.6377	246.9367	0.909698	0.362982	-259.358	708.6336
baseline_depression	-1270.83	405.9816	-3.13026	0.001747	-2066.55	-475.104
baseline_copd	-637.758	2320.614	-0.27482	0.783452	-5186.16	3910.644
baseline_asthma	-214.289	1264.464	-0.16947	0.865427	-2692.64	2264.061
baseline_ibs	1016.681	2174.717	0.4675	0.640142	-3245.76	5279.126
baseline_mx_utilisation	32.12666	11.2272	2.861502	0.004216	10.12135	54.13197
baseline_rx_utilisation	239.8731	5.851245	40.99522	0	228.4047	251.3415
year_index_date	147.8101	52.92891	2.792615	0.005228	44.06942	251.5508
DiD effect	-1745.43	244.7726	-7.13081	9.98E-13	-2225.18	-1265.67

Abbreviations: CI, confidence interval; DiD, difference-in-differences.

Table S8. Unadjusted Regression Model: Medical Costs

Model	Response_Variable	Observations	Adjusted_R_Squared		95% CI Lower Bound	95% CI Upper Bound
Unadjusted	sum_cost_imputed_inflated	27158	0.000582			
term	estimate	robust_se	t_stat	p_value		
(Intercept)	2519.5	178.6529	14.10277	0	2169.341	2869.66
sleepio_dummy	−389.886	202.3417	−1.92687	0.053996	−786.475	6.704229
post_insomnia_ sleepio_strict	256.4009	201.5556	1.27211	0.203334	−138.648	651.4498
DiD effect	−212.575	239.3971	−0.88796	0.374562	−681.794	256.6431

Abbreviations: CI, confidence interval; DiD, difference-in-differences.

Table S9. Utilization-Adjusted Regression Model: Medical Costs

Model	Response_Variable	Observations	Adjusted_R_Squared		95% CI Lower Bound	95% CI Upper Bound
Utilization-adjusted	sum_cost_imputed_inflated	27158	0.082324			
term	estimate	robust_se	t_stat	p_value		
(Intercept)	536.7075	153.2579	3.501988	0.000462	236.3219	837.0931
sleepio_dummy	67.61085	188.1194	0.359404	0.719293	−301.103	436.3248
post_insomnia_ sleepio_strict	575.6961	185.0911	3.110338	0.001869	212.9174	938.4747
baseline_mx_ utilisation	209.4442	20.49811	10.21773	0	169.2679	249.6205
baseline_rx_utilisation	−3.75528	3.240011	−1.15903	0.246443	−10.1057	2.595145
DiD effect	−321.248	226.9354	−1.41559	0.156895	−766.041	123.5457

Abbreviations: CI, confidence interval; DiD, difference-in-differences.

Table S10. Utilization, Year, and Comorbidities–Adjusted Regression Model: Medical Costs

**Model**	**Response_Variable**	**Observations**	**Adjusted_R_Squared**		**95% CI Lower Bound**	**95% CI Upper Bound**
Adjusted	sum_cost_imputed_inflated	27158	0.083013			
term	estimate	robust_se	t_stat	p_value		
(Intercept)	162504	119823.1	1.356199	0.175036	−72349.3	397357.3
sleepio_dummy	59.33248	173.2327	0.342502	0.731973	−280.204	398.8686
post_insomnia_sleepio_strict	569.1974	188.1161	3.025777	0.00248	200.4898	937.905
baseline_anxiety	−246.941	206.7308	−1.19451	0.23228	−652.134	158.2513
baseline_insomnia	−449.041	188.9208	−2.37687	0.01746	−819.326	−78.7562
baseline_diabetes	112.1882	328.3645	0.341657	0.732609	−531.406	755.7825
baseline_cardiovascular	570.3852	204.8662	2.784185	0.005366	168.8475	971.9228
baseline_depression	−656.412	240.5656	−2.72862	0.00636	−1127.92	−184.903
baseline_copd	434.0103	2062.007	0.21048	0.833293	−3607.52	4475.543
baseline_asthma	158.3589	424.1253	0.373378	0.708867	−672.927	989.6445
baseline_ibs	−369.147	773.052	−0.47752	0.632992	−1884.33	1146.035
baseline_mx_utilisation	209.3298	22.35087	9.365619	0	165.5221	253.1375
baseline_rx_utilisation	−4.26051	2.931435	−1.45339	0.146117	−10.0061	1.485108
year_index_date	−80.1868	59.28246	−1.35262	0.176176	−196.38	36.00685
DiD effect	−316.324	225.7164	−1.40142	0.161088	−758.728	126.0804

Abbreviations: CI, confidence interval; DiD, difference-in-differences.
